# Mast Cells Meet Cytomegalovirus: A New Example of Protective Mast Cell Involvement in an Infectious Disease

**DOI:** 10.3390/cells11091402

**Published:** 2022-04-21

**Authors:** Sara Becker, Matthias J. Reddehase, Niels A. Lemmermann

**Affiliations:** Institute for Virology, Research Center for Immunotherapy (FZI), University Medical Center of the Johannes Gutenberg, University Mainz, 55131 Mainz, Germany; sarbecke@uni-mainz.de (S.B.); matthias.reddehase@uni-mainz.de (M.J.R.)

**Keywords:** airway infection, antiviral protection, CCL-5 chemokine, CD8 T cells, lung infection, mast cell (MC) degranulation, pneumonia, viral mitochondria-localized inhibitor of apoptosis (vMIA)

## Abstract

Cytomegaloviruses (CMVs) belong to the β-subfamily of herpesviruses. Their host-to-host transmission involves the airways. As primary infection of an immunocompetent host causes only mild feverish symptoms, human CMV (hCMV) is usually not considered in routine differential diagnostics of common airway infections. Medical relevance results from unrestricted tissue infection in an immunocompromised host. One risk group of concern are patients who receive hematopoietic cell transplantation (HCT) for immune reconstitution following hematoablative therapy of hematopoietic malignancies. In HCT patients, interstitial pneumonia is a frequent cause of death from hCMV strains that have developed resistance against antiviral drugs. Prevention of CMV pneumonia requires efficient reconstitution of antiviral CD8 T cells that infiltrate lung tissue. A role for mast cells (MC) in the immune control of lung infection by a CMV was discovered only recently in a mouse model. MC were shown to be susceptible for productive infection and to secrete the chemokine CCL-5, which recruits antiviral CD8 T cells to the lungs and thereby improves the immune control of pulmonary infection. Here, we review recent data on the mechanism of MC-CMV interaction, a field of science that is new for CMV virologists as well as for immunologists who have specialized in MC.

## 1. Introduction

In clinical immunology, mast cells (MC) were long in the focus of attention as being cellular mediators of anaphylactic responses, allergy, and allergic asthma. These diseases are based on the release of histamine and pro-inflammatory cytokines induced by the antigen-dependent crosslinking of cognate, membrane FcεR1-bound IgE antibodies, the canonical MC receptors (for a review, see [1]). MC histamine is considered to be an axial player in stimulating the development of allergy-related inflammatory diseases by regulating the maturation and activation of leukocytes and directing their migration to target sites, where they cause chronic inflammation (reviewed in [2]). The capacity to instantly release a plethora of bioactive mediators stored in secretory granules upon signaling-induced degranulation [3] has led to the pinpointing metaphor of MC being a “loaded gun” [4]. The stimulation of MC, independently of IgE, through the ligation of pattern recognition receptors, including toll-like receptors (TLR) such as TLR3, can also induce cytokine gene expression (reviewed in [5]), including the CC chemokine ligand 5 (CCL-5, RANTES) that is known to attract T cells [6]. Of particular interest for MC function in the respiratory tract, CCL-5 drives the egress of effector T cells from the vascular compartment to the lung interstitium [7].

From an evolutionary point of view, promoting allergy in general, and specifically allergic asthma as an airway disease, is unlikely to be the physiological role of MC. These are strategically located at the body–environment interface, underneath epithelial surfaces, where they serve as frontline sentinels for invading pathogens as part of the immune defense against infections. They may be viewed as being “inbetweeners”, involved in both innate and adaptive immunity [8,9]. As mucosal surfaces, particularly those of the airways, are the natural entry sites for many types of viruses, viral infections are likely to reveal the physiological role of MC (for a recent review, see [10]). With a focus on in vivo models, Table 1 compiles studies on the role of MC in virus infections. In essence, this role is ambiguous in that MC can contribute to the control of infection but, in some cases, were also reported to exacerbate viral disease. Examples include Dengue Virus, Influenza A Virus, Japanese Encephalitis Virus, and SARS-CoV-2 [11,12,13,14].

Here, we review data, adding cytomegaloviruses (CMVs) to the list as an example for a beneficial role of MC through their contribution to the prevention of interstitial CMV pneumonia.

## 2. Cytomegalovirus Pathogenesis and Its Relation to Airway Infection

Human cytomegalovirus (hCMV) is the prototype member of the β-subfamily of the herpes virus family [35]. Host-species restriction of CMVs precludes studying hCMV in experimental animal models. Clinical investigation of hCMV in its human host is limited since ethical standards prohibit targeted mutations of virus and host for experimental approaches. As we have reviewed previously [36], biological convergence in the adaptation of the virus to its specific host during co-evolution has led to common principles of viral pathogenesis and host immune response across different virus–host pairs. So, specifically, the mouse model, based on infection with murine CMV (mCMV), has proven its predictive value for human CMV disease and immune control in many aspects.

As a common feature of all CMVs, primary infection of an immunocompetent host is efficiently controlled by mechanisms of innate and adaptive immunity, so that primary infection of humans, which occurs mostly in early childhood, passes unnoticed with no overt clinical symptoms, and is thus rarely diagnosed. The virus, however, is not cleared but is maintained lifelong in certain cell types in a latent stage, known as “latency” (for a review, see [37]). Latency is defined by the absence of the infectious virus, despite the presence of the viral genome, from which genes can be expressed stochastically without completing the productive viral replication cycle but with profound effects on cellular gene expression in latently infected host cells and on their immune surveillance [38,39,40,41,42,43].

Clinical relevance of hCMV results from cytopathogenic tissue infection leading to birth defects in congenitally infected immunologically immature fetuses of expectant mothers undergoing primary infection or productive reactivation of latent infection [44,45], as well as from unrestricted tissue infection resulting in CMV disease with multiple organ failure in immunocompromised hosts [46,47]. A risk group of concern, at transplantation centers worldwide, are recipients of hematopoietic cell transplantation (HCT). In these patients, who are transiently immunocompromised in the phase of ongoing hematopoietic reconstitution, latent virus can reactivate either from transplanted cells or from organs of the recipient, or both [37,48,49,50]. This bears a high risk of developing lethal interstitial pneumonia [51,52,53,54], in particular, after histoincompatible, allogeneic HCT (for a review, see [55]) and when the infection is by virus strains that resist standard antiviral chemotherapy [56,57,58]. In accordance with clinical manifestations of hCMV infection, the mCMV model has identified a prominent role of lung infection in viral pathogenesis. Upon acute infection of neonatal mice, the lungs were shown to represent an organ site of viral pathogenesis, involving the infection of alveolar macrophages [59]. After clearance of acute neonatal infection, the lungs were found to harbor a particularly high load of latent virus, resulting in a high incidence of recurrent productive infection upon immunosuppression [60]. In the immunocompromised host [61], as well as in experimental HCT recipients [62,63], interstitial pneumonia was shown to be a paramount manifestation of CMV disease with a high risk of a lethal outcome.

Infection with hCMV is not usually taken into consideration as a respiratory infection in childhood, and hCMV is therefore not included in diagnostic panels. Thus, primary infection is rarely diagnosed. However, host-to-host CMV transmission occurs through saliva by smear infection, as well as droplet inhalation. Thereby, the virus reaches the mucosal surfaces of the upper and lower airways [64,65,66]. As we have shown in the mouse model, mCMV airway infection can promote allergic airway disease by activating migratory dendritic cells [67,68], and airway challenge infection of latently infected mice can recruit effector-memory CD8 T cells from the vascular compartment to the alveolar epithelium [69]. It is, thus, more than likely that CMVs meet MC at respiratory tract surfaces and that MC do not remain uninvolved.

## 3. Infection Induces a Serum Wave of Chemokine CCL-5 in MC-Competent Mice

A previous study on influenza and parainfluenza virus infections of the respiratory tract revealed the importance of chemokine receptor CCR-5 expression on CD8 T cells for accelerated recruitment to the lungs [70], although the source of the ligand CCL-5 and a possible contribution of MC was not specified. More directly, a role for MC-derived CCL-5 was shown for the airway recruitment of CD8 T cells in the respiratory infection with Newcastle disease virus (Table 1, [6]). Consistent with this, a cell culture model of reovirus infection of human MC revealed chemotaxis of human CD56^+^ T cells, depending on the secretion of CCR ligands [71].

**Figure 1:**
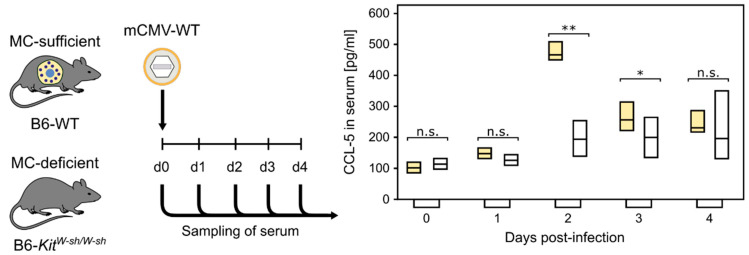
MC-dependent wave of serum chemokine CCL-5. (n.s.) Not significant; (*) *p* < 0.05; (**) *p* < 0.01. (Yellow bars) Presence of MC. (Open bars) Absence of MC. Bars represent the range of data from individual mice. Median values are indicated. Results are displayed schematically based on original data published in [73].

Our previous study, comparing acute, primary mCMV infection in MC-competent C57BL/6 (B6) wild-type mice and MC-deficient mutants C57BL/6 (B6)-*Kit^W-sh/W-sh^* (briefly *sash* mutants) [72], showed a strong MC-dependent wave of serum CCL-5 peaking on day two, post-infection (Figure 1, [73]). CCL-5 is not contained in MC granules and, thus, its release requires new synthesis [3].

## 4. MC Recruit CD8 T Cells to the Lungs for an Enhanced Control of Infection

**Figure 2:**
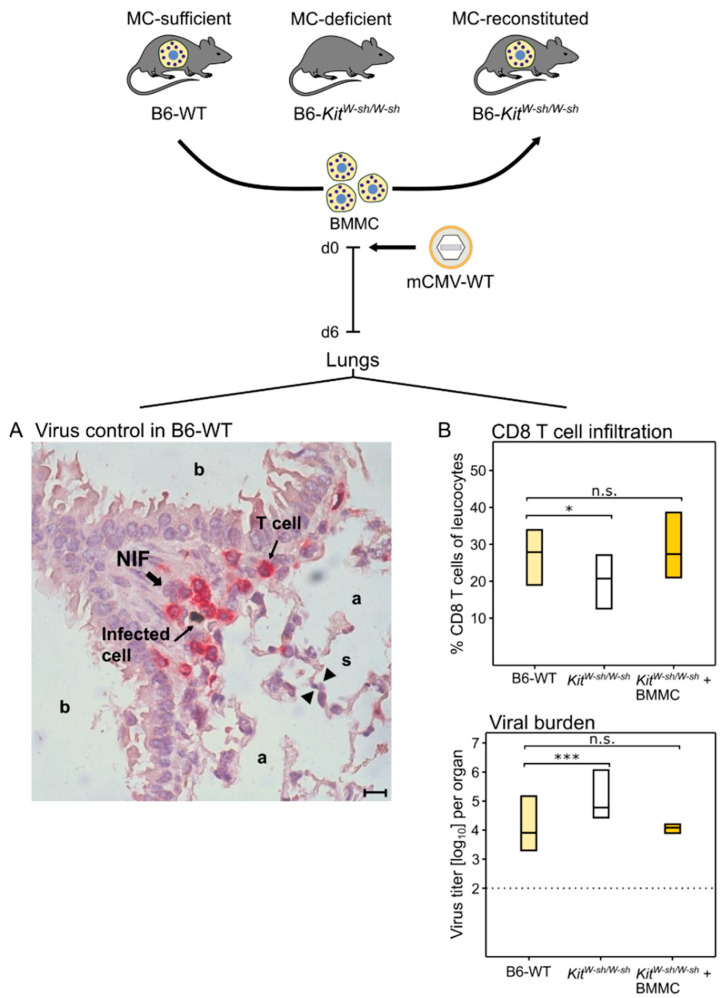
(Top) Experimental protocol. (BMMC) Bone-marrow-derived MC. MC-deficient mice were reconstituted by intravenous infusion of BMMC derived from MC-sufficient donors. Experiments were performed 4 weeks after engraftment. (**A**) Two-color immunohistological image showing the confinement of lung infection by CD8 T cells in a nodular inflammatory focus (NIF) on day six after intravenous infection of MC-sufficient B6-WT (wild-type) mice; (a) alveoli lined with alveolar epithelium; (b) bronchioles lined with bronchiolar epithelium; and (s) alveolar septum. The bar marker represents 10 μm. (**B**) Inverse correlation between CD8 T-cell tissue infiltration and control of lung infection. Bars represent the range of data from individual mice with the median values indicated. (Light yellow bars) Constitutively MC-competent B6-WT mice; (open bars) MC-deficient *sash* mutants; (dark yellow bars) MC-deficient *sash* mutants made MC-competent by reconstitution with BMMC. (n.s.) Not significant; (*) *p* < 0.05; (***) *p* < 0.001. Results are displayed schematically and in a new arrangement based on original data published in [73].

As CCL-5 is known to recruit antiviral CD8 T cells to the lungs (see above, [6,70]), this was predicted to apply, also, to pulmonary CD8 T-cell infiltration in the course of an acute CMV infection of the lungs. The immunocompetent mouse model of intravenous infection with mCMV indeed revealed that the wave of MC-dependent CCL-5 (Figure 1) is followed by lung infiltration with antiviral CD8 T cells that confine and eventually resolve tissue infection within nodular inflammatory foci (NIF) ((Figure 2A), [73]).

NIF consist primarily of tissue-infiltrating CD8 T cells that are not distributed randomly but cluster around infected cells [63] depending on the presentation of cognate viral epitopes [74,75]. Lung infiltration by CD8 T cells and lung infection were found to be inversely correlated (Figure 2B, [73]), reflecting the fact that CD8 T cells represent the antiviral effector cells in pulmonary infiltrates [61,62,63]. Specifically, a role for MC was shown by reduced pulmonary CD8 T-cell infiltration corresponding to reduced control of pulmonary infection in MC-deficient *sash* mutants compared with MC-competent B6-WT (wild-type) mice. Importantly, lung infiltration by CD8 T cells and control of infection were restored when MC-deficiency was reversed by the reconstitution of *sash* mice with bone-marrow-derived MC (BMMC) from B6-WT donors (Figure 2B, [73]).

## 5. MC Are Targets of Productive In Vivo Infection

As shown by Matsuma and colleagues [76], BMMC are not a reliable cell culture model for MC functions in vivo in the context of tissues, which can be explained by different stages of maturation and pre-activation. Whereas standard cell-culture BMMC were at first found to be refractory to mCMV infection [73], a subsequent study revealed that BMMC, activated by the Ca^2+^ ionophore ionomycin, are permissive to productive mCMV infection [77]. As ionomycin rapidly raises the intracellular level of Ca^2+^, this finding gave a first hint to the critical role of Ca^2+^ mobilization in the infection of MC by mCMV.

**Figure 3:**
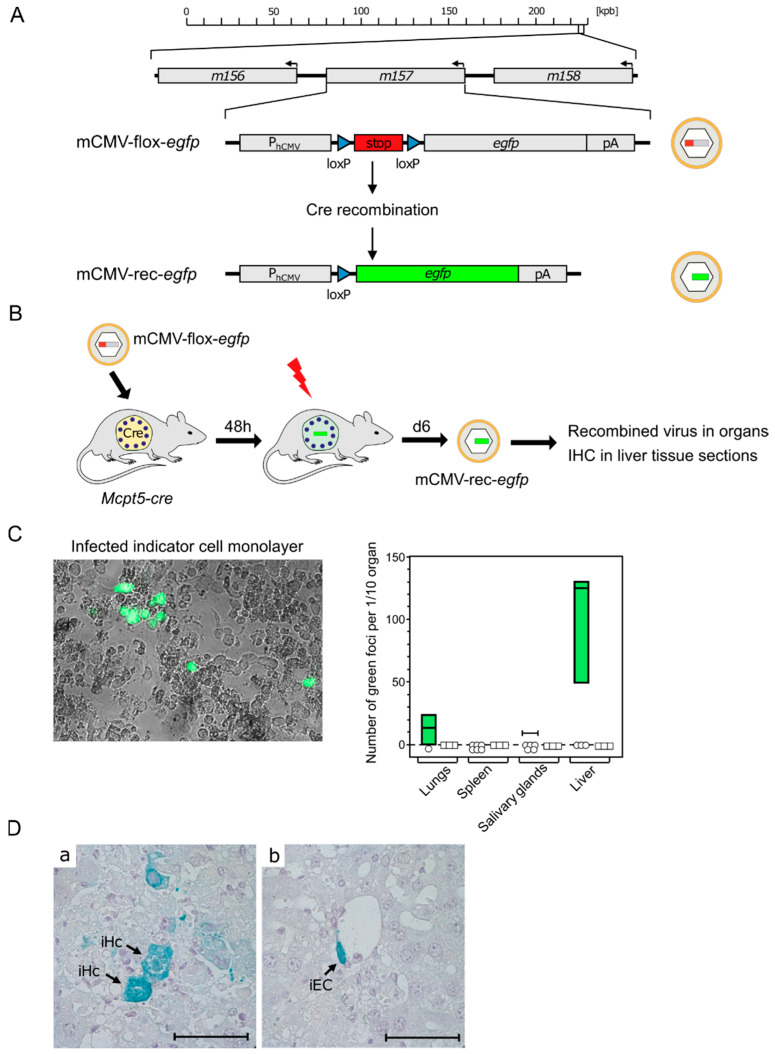
Evidence for productive mCMV infection of MC in vivo. (**A**) Gene maps explaining the principle of Cre recombination for generating reporter virus mCMV-rec-*egfp*. (kbp) Kilobase pairs of the linear, double-stranded DNA genome of mCMV. It circularizes in the infected cell and does not integrate into the cellular genome. The genomic region of interest is shown in detail. Arrows indicate the direction of transcription. (P_hCMV_) hCMV promoter–enhancer; (pA) polyadenylation. (**B**) Experimental protocol. *Mcpt5-cre* mice were infected intraperitoneally with virus mCMV-flox-*egfp*. (Flash symbol) Total-body γ-irradiation with a dose of 7 Gy for immunosuppression to facilitate in vivo spread of recombined virus mCMV-rec-*egfp*. (**C**) Infection of mouse embryo fibroblast (MEF) monolayer cultures by homogenates of the indicated organs taken on day eight after infection. (Left) eGFP fluorescence and phase-contrast microscopy image showing green-fluorescent MEF infected with MC-derived mCMV-rec-*egfp*. (Right) Quantitation of green foci of MEF infected with MC-derived mCMV-rec-*egfp*. Green bars represent range, with median values marked, of data only from individual mice in which recombination has occurred to yield green-fluorescent MEF. Open symbols indicate mice in which recombinations were not detected in the respective organ. (**D**) Detection of infected cells in liver tissue sections taken on day eight after infection. Cells expressing MC-derived mCMV-rec-*egfp* were stained in turquoise color by immunohistochemistry (IHC) specific for eGFP. Light hematoxylin counterstain. The bar marker represents 50 μm. Image: (**a**) iHc, infected hepatocytes; and (**b**) iEC, infected vascular endothelial cell. Results are displayed schematically and in a new arrangement based on original data published in [80] (**C**) and in [81] (**D**).

Although results from BMMC in cell culture can provide insights into MC-CMV interactions, the in vivo function of MC is what actually counts for the role of MC in viral pathogenesis and immune control. For testing the permissivity of tissue-resident MC to mCMV infection, we made use of the recombinant virus mCMV-flox-*egfp* [78] (Figure 3A) and of transgenic *Mcpt5-cre* mice expressing Cre recombinase under the control of the MC-specific protease 5 promoter [79]. Upon the infection of *Mcpt5-cre* mice with mCMV-flox-*egfp*, the floxed stop cassette is cleaved out by Cre recombinase selectively in MC. This results in recombined virus mCMV-rec-*egfp*, from which enhanced green fluorescent protein (eGFP) can be expressed in infected MC as well as in secondary target cells, provided that infection of MC is productive and leads to the release of the recombined virus (Figure 3B).

To identify and quantitate infectious mCMV-rec-*egfp*, organ homogenates were plated on monolayers of permissive mouse embryo fibroblasts as indicator cells and, after a period in cell culture, indicator cells expressing the reporter protein eGFP were counted (Figure 3C) [80]. The finding that the liver was the predominant source of the recombined virus is explained by the fact that the hepatocyte is the major virus-producing cell type during acute mCMV infection [78]. The existence of green-fluorescent indicator cells proved that mCMV-flox-*egfp* was recombined in MC, and that the viral replication cycle has proceeded within MC to the assembly of infectious mCMV-rec-*egfp* virions.

Finally, it remained to clarify the question if recombined reporter virus, isolated from infected organs, localized to tissue-resident MC only, or if the reporter virus was released from productively infected MC and spread to other cell types. As shown by the immunohistochemical (IHC) detection of eGFP, for the example of the liver, the virus produced in MC spread, indeed, to other cell types, such as hepatocytes and vascular endothelial cells (Figure 3D) [81]. Although infection of MC is productive, MC-derived virus is unlikely to contribute significantly to viral pathogenesis compared with many other cell types with much higher virus productivity [78].

## 6. Expression of a Viral Mitochondria-Localized Inhibitor of Apoptosis (vMIA) Is Critical for MC Degranulation

**Figure 4:**
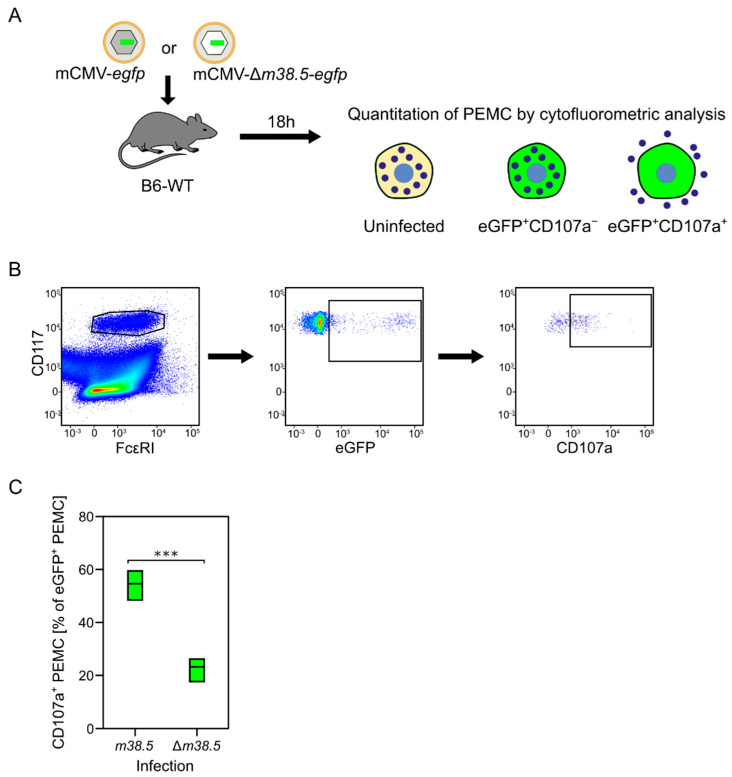
MC degranulation triggered by the anti-apoptotic mCMV protein vMIA-m38.5. (**A**) Sketch of the experimental protocol. C57BL/6 mice were infected intraperitoneally with mCMV recombinants containing (grey-shaded capsid) or lacking (empty capsid) gene *m38.5*. Both virus genomes include gene *egfp* coding for the fluorescent reporter protein eGFP (green) that identifies infected MC. Peritoneal exudate MC (PEMC) were isolated from the peritoneal cavity 18 hours (h) after infection. (**B**) Gating strategy for cytofluorometric analysis identifying CD117^+^FcεR1^+^ PEMC that either have remained uninfected (eGFP^−^) or were infected but have not, or not yet, degranulated (eGFP^+^CD107a^−^), or were infected and have already degranulated (eGFP^+^CD107a^+^). (**C**) Relative quantitation of infected eGFP^+^CD117^+^FcεRI^+^CD107a^+^ PEMC degranulated after infection with either mCMV-*egfp* expressing vMIA-m38.5 or deletion mutant mCMV-Δm36.5-*egfp*. Green bars represent range of data from individual mice, with median values marked. (***) highly significant with *p* < 0.001. Results are displayed graphically modified and in a new arrangement, based on original data published in [77].

Our previous study identified two waves of degranulation of CD117^+^FcεRI^+^ peritoneal exudate MC (PEMC) upon the indirect or direct effects of mCMV. A first wave at 4 h after intraperitoneal infection was found to be toll-like receptor 3 (TLR3)-dependent and involve an, as yet, unidentified other cell type. In contrast, a later wave at 24 h proved to be independent of TLR3-TRIF signaling, but dependent on MC infection [80]. As the MC-derived CCL-5 has its serum peak on day two (Figure 1), and as MC-enhanced control of lung infection was still operative in TLR3 knock-out mice [82], our interest turned to MC degranulation triggered by the infection of MC. Recent work has identified the expression of the anti-apoptotic mCMV protein vMIA-m38.5 as being critical for MC activation to degranulation [77] (Figure 4).

MC have long served as a preeminent model for studying Ca^2+^-dependent exocytosis. Basic features are the release of Ca^2+^ from Ca^2+^ stores in the endoplasmic reticulum (ER), the coupling of ER store depletion to influx of external Ca^2+^, and the subsequent uptake of excess Ca^2+^ into ER and mitochondria through ATP-dependent Ca^2+^ pumps (for a review, see [83]). Our own study has revealed a link between Ca^2+^ mobilization and the permissivity of MC to infection with mCMV [77]. As the hCMV protein vMIA-pUL37x1 has been shown to release Ca^2+^ from ER stores [84], we analyzed the role of its mCMV analog vMIA-m38.5 [85,86,87,88] in the degranulation of PEMC recovered from C57BL/6 mice infected intraperitoneally with either mCMV-*egfp*, expressing protein vMIA-m38.5, or with the gene deletion mutant mCMV-Δ*m38.5*-*egfp* [77]. The results of this approach (Figure 4) and of the ex vivo infection of PEMC, as well as from the transfection of BMMC with gene *m38.5* [77], consistently revealed that vMIA-m38.5 induces MC degranulation.

## 7. Synopsis, Open Questions, and Outlook

**Figure 5:**
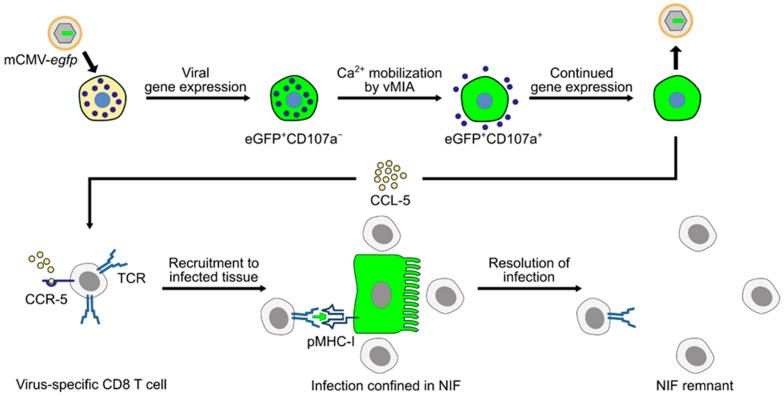
Graphical abstract summarizing the current knowledge regarding the role of MC in controlling CMV infection in the mouse model of CMV disease. Reporter protein eGFP (green color) is used to indicate infection. (TCR) T-cell receptor; (pMHC-I) MHC class-I molecule presenting viral peptide to cognate CD8 T cells; (NIF) nodular inflammatory focus; and (vMIA) viral mitochondria-localized inhibitor of apoptosis.

The picture that has now emerged is that mCMV productively infects MC in vivo and that expression of the anti-apoptotic mCMV protein vMIA-m38.5 activates MC for degranulation, most likely involving Ca^2+^ mobilization. Infected MC secrete chemokine CCL-5, which recruits virus-specific CD8 T cells to infected organs, particularly to the lungs, resulting in enhanced antiviral control and prevention of interstitial CMV pneumonia, as well as other manifestations of CMV disease (Figure 5).

Research into the molecular mechanisms of CMV-MC interaction and into the role MC play in CMV pathogenesis or immune control is still far from an end. There exist open questions that need to be addressed in future work. MC-derived CCL-5 appears to be critically involved in attracting antiviral CD8 T cells to sites of infection, specifically to the lungs, but it is not contained in the granules [3] and is thus not released upon MC degranulation. Molecular evidence for an enhanced CCL-5 gene expression in infected cells is still missing, and a possible role for a virally encoded transcriptional transactivator awaits analysis. If degranulation of MC in response to Ca^2+^ mobilization by vMIA-m38.5 plays any role at all is an enthralling issue. To return to the title of this article, another knowledge gap concerns the in vivo site at which “mast cells meet cytomegalovirus” physically, since infected and degranulated MC were not yet shown in airway mucosa after airway infection or after intravenous infection. Finally, one would like to see if key findings of the mouse model apply also to hCMV and human MC, at least in cell culture.

Over decades, the main interest of our group has been using the mouse model for a better understanding of CMV interstitial pneumonia and its prevention in human HCT recipients (reviewed in [36,49,55]). A possible involvement of MC in the reconstitution of protective antiviral immunity against hCMV reactivation after HCT has never even been considered in clinical HCT. Work is in progress to evaluate the contributions of donor-genotype MC that become reconstituted by hematopoiesis and of tissue-resident recipient-genotype MC that are resistant to the hematoablative treatment. It is hoped that supplementation of the hematopoietic stem- and progenitor-cell transplant with mature MC improves the recruitment of endogenously reconstituted, or of adoptively transferred, antiviral CD8 T cells to the lungs for more efficiently controlling, and hopefully preventing, interstitial CMV pneumonia.

## Figures and Tables

**Table 1:** Involvement of MC in control or exacerbation of viral infections. * MC-deficient mouse strain.

Virus	Route of Host Infection	MC Subpopulation	Animal Model	Response of MC	Proposed Role of MC	Ref.
Dengue Virus	Mosquito-borne	CD117 (c-kit)	* Kit^W-sh/W-sh^	Degranulation Production of cytokines/chemokines, chymase and leukotrienes,	Recruitment of NK and NKT cells; Recruitment of macrophages; Activation of γδ T cells Increased vascular leakage	[11,15,16,17,18]
Herpes Simplex Virus 1	Mucosal		* Kit^W-sh/W-sh^		Regulate tissue infiltration of neutrophils in the cornea	[19]
Herpes Simplex Virus 2	Mucosal		* Kit^W/Wv^	Release of TNF and IL-6, but no degranulation		[20,21]
Influenza A Virus	Respiratory	MC progenitors: CD45^+^; Lin^−/lo^; CD117^hi^ (c-kit^hi^); FcεRI^+^; CD16/32^int^; integrin β7^hi^	* Kit^W-sh/W-sh^	Degranulation Cytokine/chemokine, histamine and tryptase release, leukotriene production		[12,22,23,24]
Japanese Encephalitis Virus	Mosquito-borne		* Kit^W-sh/W-sh^	Degranulation	Increased leakage of blood-brain barrier leads to enhanced infection of the central nervous system	[13]
Lymphocytic Choriomeningitis Virus	Intradermal		*MasTRECK		Activation of DCs and splenic macrophages	[25]
Newcastle Disease Virus	Respiratory	CD117^+^ (c-kit^+^), FcεRI^+^, S/T2^+^	* Kit^W/Wv^	Release of MIP1-B and CCL-5	Recruitment of CD8 T cells	[6]
Parainfluenza Virus 3	Respiratory		Guinea pig	Degranulation Histamine release		[26]
Respiratory Syncytial Virus	Respiratory		Bovine	Release of LL-37 (antimicrobial peptide)		[27]
SARS-CoV-2	Respiratory		Humanized mice; Rhesus macaques	Degranulation Upregulation of cytokines/chemokines, and metallopeptidases		[14]
Sendai Virus	Droplet/respiratory		Rat	Histamine release		[28,29]
Sindbis Virus	Mosquito-borne		* WBB6F1-W/Wv		Facilitate entry of inflammatory cells into central nervous system	[30]
Vaccinia Virus	Intradermal		* Kit^W-sh/W-sh^	Degranulation Release of LL-37 (antimicrobial peptide)		[31,32,33]
Vesicular Stomatitis Virus	Mosquito-borne, droplet		* Kit^W-sh/W-sh^	Release of type I IFN, IL-6, IP-10, MCP1, MIP1β		[34]

## Data Availability

The data presented in this study are available on request from the corresponding author.
